# Anticoagulants for Stroke Prevention in Atrial Fibrillation in Elderly Patients

**DOI:** 10.1007/s10557-020-06981-3

**Published:** 2020-04-29

**Authors:** Andreas Schäfer, Ulrike Flierl, Dominik Berliner, Johann Bauersachs

**Affiliations:** Department of Cardiology and Angiology, Hannover Medical School, Carl-Neuberg-Str. 1, D-30659 Hannover, Germany

**Keywords:** Anticoagulation, VKA, NOAC, Elderly patients, Atrial fibrillation

## Abstract

Ischaemic stroke and systemic embolism are the major potentially preventable complications of atrial fibrillation (AF) leading to severe morbidity and mortality. Anticoagulation using vitamin K antagonists (VKA) or non-vitamin K oral anticoagulants (NOACs) is mandatory for stroke prevention in AF. Following approval of the four NOACs dabigatran, rivaroxaban, apixaban, and edoxaban, the use of VKA is declining steadily. Increasing age with thresholds of 65 and 75 years is a strong risk factor when determining annual stroke risk in AF patients. Current recommendations such as the “2016 Guidelines for the management of atrial fibrillation” of the European Society of Cardiology and the “2019 AHA/ACC/HRS Focused Update” by the American College of Cardiology, the American Heart Association, and the Heart Rhythm Society strengthen the importance of anticoagulation and detection of bleeding risks, of which older age is an important one. While patients aged ≥ 75 years are usually underrepresented in randomised clinical trials, they represent almost 40% of the trial populations in the large NOAC approval studies. Therefore, a sufficient amount of data is available to assess the efficacy and safety for this patient cohort in that specific indication. In this article, the evidence for stroke prevention in AF using either VKA or NOACs is summarised with a special focus on efficacy compared to bleeding risk in patients aged ≥ 75 years. Specifically, we used a model of increased weighing of intracranial bleeding to illustrate the potential benefit of NOACs over VKA in the elderly population. In brief, there are at least two tested strategies with apixaban and edoxaban which even confer an additional clinical net benefit compared with VKA. Furthermore, elderly subgroups of trials for combined antithrombotic treatment following percutaneous coronary interventions in anticoagulated patients are analysed.

## Indication for Stroke Prevention in Elderly Patients

Ischaemic or embolic strokes are the major disabling complications in patients with atrial fibrillation (AF). Oral anticoagulation (OAC) can prevent most of these events and is, therefore, widely used for stroke prevention in AF. Excluding patients with very low stroke risks, the superiority of OAC compared with non-treatment is overwhelming and, based on current European Society of Cardiology (ESC) “2016 Guidelines for the management of atrial fibrillation” and the “2019 AHA/ACC/HRS Focused Update of the 2014 Guideline for the Management of Patients With Atrial Fibrillation” by the American College of Cardiology, the American Heart Association, and the Heart Rhythm Society, should be used in stroke prevention in AF [1, 2]. Furthermore, the European Heart Rhythm Association (EHRA) “2018 EHRA Practical Guide on the use of non-vitamin K antagonist oral anticoagulants in patients with atrial fibrillation” elucidates the advantage of non-vitamin K oral anticoagulants (NOACs) compared with vitamin K antagonists (VKA) [3]. Previously, a potential benefit of certain NOACs compared with VKA has been described in a meta-analysis including differing indications for anticoagulation [4]. This article will focus in detail on patients ≥ 75 years of age treated for stroke prevention in AF. This threshold alone indicates higher embolic as well as increased bleeding risk when using contemporary scoring tools [5, 6].

In principle, guidelines recommend stroke prevention in AF using OAC in all patients with CHA_2_DS_2_-VASc score ≥ 2 in men and ≥ 3 in women. Since age ≥ 75 years gives 2 score points and female sex 1 score point, all patients ≥ 75 years of age are recommended to receive OAC with a class Ia recommendation irrespective of the presence or absence of additional risk factors [1, 2]. While previous meta-analyses focussed on elderly patients in phase II and III trials in AF and venous thromboembolism [4, 7], the current manuscript focuses on patients ≥ 75 years of age with a specific consideration of intracranial bleeding-related disability and on contemporary data from trials combining OAC with antiplatelet agents following coronary interventions in anticoagulated AF patients.

## Anticoagulation or Antiplatelet Therapy for Stroke Prevention in Atrial Fibrillation in Elderly Patients

Antiplatelet therapy using acetylsalicylic acid had been a low level recommendation in AF patients with CHA_2_DS_2_-VASc score = 1 in previous guidelines, but no longer in the current European or American guidelines [1, 2]. In the European guidelines, it is clearly stated that “antiplatelet monotherapy is not recommended for stroke prevention in AF patients, regardless of stroke risk”. In the past, acetylsalicylic acid had been considered as a less harmful option in patients deemed not suitable for OAC due to higher bleeding complications especially in elderly patients. However, in primary prevention trials for cardiovascular disease, age ≥ 70 years was associated with significantly increased bleeding rates on acetylsalicylic acid compared with younger individuals [8, 9]. In the Oxford Vascular Study performed in patients with clinically evident atherosclerosis, an increase of major bleedings on acetylsalicylic acid to an annual rate of 2% in patients aged 75–84 years and of 4% in patients above 84 years had been observed; life-threatening or fatal bleedings ranged between 1.0 and 2.5% annually [10]. The findings regarding acetylsalicylic acid are reproducible in several trials with patients ≥ 75 years of age; similarly the rate of major bleeds was 5.2%/year in the recent RE-SPECT ESUS trial [11]. In the Birmingham Atrial Fibrillation Treatment of the Aged Study, patients ≥ 75 years of age on acetylsalicylic acid did not show a lower rate of major bleedings compared with VKA, but VKA was superior to acetylsalicylic acid regarding stroke prevention. In this study, even very elderly patients, which are often categorised as frail, had a 50% risk reduction for embolic/ischaemic events on VKA with a similar bleeding risk as on acetylsalicylic acid [12]. However, it has to be considered that VKA treatment in this trial was well controlled with time in therapeutic range of 67%. In the AVERROES trial comparing acetylsalicylic acid and the NOAC apixaban, bleeding rates in elderly AF patients were similarly increased as in the Oxford Vascular Study when treated with acetylsalicylic acid as monotherapy indicating that the observed bleeding rates were representative for this patient population. Of note, when patients were above 85 years, annual rates for stroke or systemic embolism increased to 6.5%, for major bleedings to 4.7%, and for intracranial haemorrhage to 2.9% on acetylsalicylic acid, but anticoagulation with apixaban showed significantly lower rates of stroke or systemic embolism with safety comparable to acetylsalicylic acid [13].

The four randomised controlled trials evaluating NOACs compared with VKA for stroke prevention in AF consistently showed an annual major bleeding rate of 4.4–5.2% on VKA in AF patients ≥ 75 years of age (Table 1) [14–17]. When balancing the rates of stroke or systemic embolism on VKA to that of intracranial haemorrhage, the strongest clinical net benefit is in patients at greatest risk, e.g. those above 85 years of age [18]. In general, while the bleeding rate in elderly patients might be lower on acetylsalicylic acid than on VKA, there is still a much stronger efficacy with VKA. Considering the clinical net benefit, acetylsalicylic acid is not an option for stroke prevention in AF in patients ≥ 75 years of age because of comparable harm but much lower efficacy. Therefore, the contraindication from current guidelines regarding antiplatelet monotherapy for stroke prevention in AF also applies for elderly patients, irrespective whether a population above 75 or above 85 years of age is considered. The combination of NOACs and platelet inhibitors in elderly patients was beyond the scope of this review and has recently been reviewed [19].

**Table 1:** Characteristics of NOAC approval trials

	RE-LY [15]	ROCKET-AF [16]	ARISTOTLE [17]	ENGAGE-AF [14]
Comparator to VKA	Dabigatran	Rivaroxaban	Apixaban	Edoxaban
Standard dose	150 mg twice daily 110 mg twice daily	20 mg once daily	5 mg twice daily	60 mg once daily
Reduced dose	–	15 mg once daily	2.5 mg twice daily	30 mg once daily
Dose reduction criteria	1:1:1 randomised to either dose or VKA	Creatinine clearance 30–49 ml/min	At least two criteria: • Age ≥ 80 years • Body weight < 60 kg • Serum creatinine ≥ 1.5 mg/dL	at least one criterion: • Estimated creatinine clearance 30–50 ml/min • Body weight ≤ 60 kg • Concomitant use: ciclosporine, dronedarone, erythromycin, or ketoconazole
Patients (*n*)	18,113	14,264	18,201	14,071
Patients with dose reduction criteria	N/A	2950 (21%)	831 (5%)	5356/21,105 (25%)
Average age (years)	72	73	70	72
≥ 75 years	7258 (40%)	6229 (44%)	5678 (31%)	5668 (40%)
Patients with dose reduction criteria ≥ 75 years	N/A	2272 (36%)	Patients with dose reduction ≥ 75 years	N/A
CHADS_2_	2.1 ± 1.1	3.5 ± 0.9	2.1 ± 1.1	2.8 ± 1.0
≥ 75 years	N/A	3.7 ± 1.0	2.7 ± 1.1	3.2 ± 1.1
Predicted stroke rate for patients ≥ 75 years based on CHADS_2_ (%/year)	~ 4	6–8	4–6	~ 6
Overall relative risk vs. VKA for stroke/systemic embolism, RR (95% CI)	110 mg, 0.91 (0.74–1.11) 150 mg, 0.66 (0.53–0.82)	0.88 (0.75–1.03)	0.79 (0.66–0.95)	0.87 (0.73–1.04)
Overall relative risk vs. VKA for primary safety, RR (95% CI)	110 mg, 0.80 (0.69–0.93) 150 mg, 0.93 (0.81–1.07)	1.03 (0.96–1.11)	0.69 (0.60–0.80)	0.80 (0.71–0.91)
Presence of stroke risk factors in patients ≥ 75 years at baseline	Data not reported	C:58.6% H:92.7% A:100% D:33.8% S:41.6%	C: 24.3% H: 83.0% A: 100% D: 21.1% S: 21.8%	C: 45% H: 93% A: 100% D: 28% S: 25%
Renal function in patients ≥ 75 years at baseline	Creatinine clearance • ≥ 80 ml/min: 12% • 50–79 ml/min: 57% • < 50 ml/min: 26%	Creatinine clearance, median 55 ml/min (IQR 44, 68)	Creatinine clearance • > 80 ml/min: 10.5% • 51–80 ml/min: 51.5% • 31–50 ml/min: 33.6% • ≤ 30 ml/min: 3.9%	Creatinine clearance • > 80 ml/min: 12% • 51–80 ml/min: 52% • ≤ 50 ml/min: 37%
Definition of primary safety endpoint	Major bleeding defined as a reduction in the haemoglobin level of at least 2 g/dL, transfusion of at least 2 units of blood or requiring inotropic agents, symptomatic bleeding in a critical area or organ	Composite of major and non-major clinically relevant (NMCR) bleeding: • Major bleeding was defined as clinically overt bleeding associated with any of the following: fatal outcome, involvement of a critical anatomic site (intracranial, spinal, ocular, pericardial, articular, retroperitoneal, or intramuscular with compartment syndrome), fall in haemoglobin concentration > 2 g/dL, transfusion of > 2 units of whole blood or packed red blood cells, or permanent disability • NMCR bleeding was defined as overt bleeding not meeting criteria for major bleeding but requiring medical intervention, unscheduled contact (visit or telephone) with a physician, temporary interruption of study drug (i.e. delayed dosing), pain, or impairment of daily activities	Major bleeding defined by ISTH criteria: • Fatal bleeding • Symptomatic bleeding in a critical area or organ such as intracranial, intraspinal, intraocular, retroperitoneal, intra-articular or pericardial, or intramuscular with compartment syndrome • Bleeding causing a fall in haemoglobin level ≥ 2 g/dL or leading to transfusion ≥ 2 units of whole blood or red cells	Major bleeding defined by ISTH criteria: • Fatal bleeding • Symptomatic bleeding in a critical area or organ such as intracranial, intraspinal, intraocular, retroperitoneal, intra-articular or pericardial, or intramuscular with compartment syndrome • Bleeding causing a fall in haemoglobin level ≥ 2 g/dL or leading to transfusion ≥ 2 units of whole blood or red cells

Dosing, dose reduction criteria, predicted stroke risk, and proportion of patients with age ≥ 75 years in the four trials comparing non-vitamin K oral anticoagulants to vitamin K antagonist for stroke prevention in atrial fibrillation. VKA, vitamin K antagonist; *for the RE-LY trial only the mean CHADS_2_ score independent from age was reported in the publication about influence of age on bleeding rates [20]

## NOACs for Stroke Prevention in AF in the Elderly: Approval Trials

As the four different studies evaluating NOACs compared with VKA for stroke prevention in AF are different in inclusion and exclusion criteria as well as in underlying stroke risk by CHADS_2_ score, the 4 NOACs cannot be compared directly, but an impression on clinical net benefit consisting of the trials individual primary efficacy (usually stroke or systemic embolism) and safety (major bleedings according to study definition) endpoints can be given. The different dosing regimens, bleeding definitions, and proportion of elderly within the trial as well as the effect on primary endpoints in the overall populations are summarised in Table 1, while the main text refers to relative increase and decrease of events in the studies.

### Dabigatran in RE-LY

**Fig. 1:**
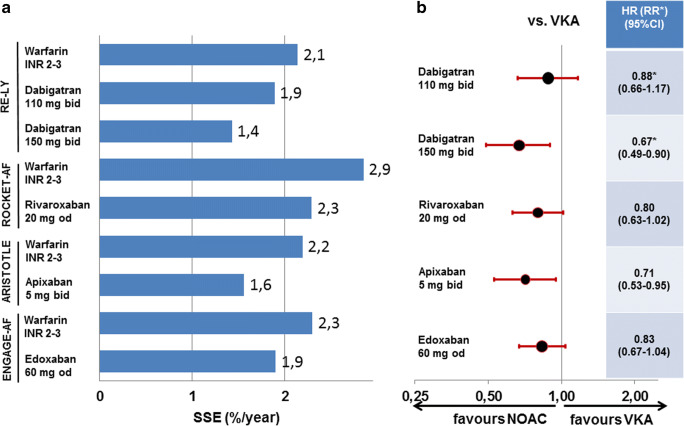
Event rates (**a**) and hazard ratios (or relative risk for dabigatran) (**b**) for stroke or systemic embolism in patients ≥ 75 years of age in the four trials comparing non-vitamin K oral anticoagulants to vitamin K antagonist for stroke prevention in atrial fibrillation. HR, hazard ratio; NOAC, non-vitamin K oral anticoagulants; RR, relative risk; SSE, stroke or systemic embolism; VKA, vitamin K antagonist. Definition of major bleeding according to study criteria as well as dosing regimens is given for all studies in Table 1

**Fig. 2:**
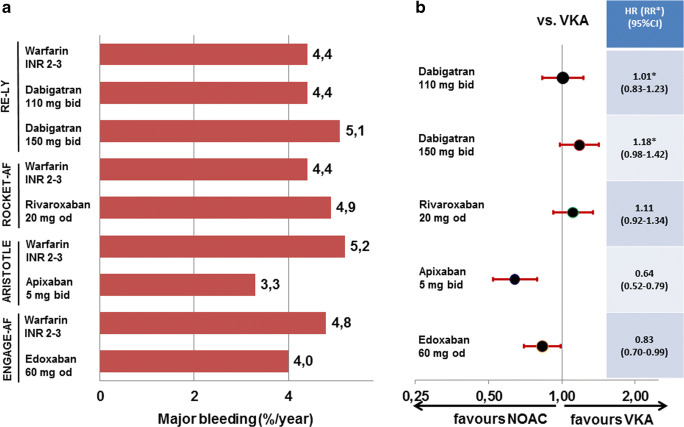
Event rates (**a**) and hazard ratios (or relative risk for dabigatran) (**b**) for major bleedings in patients ≥ 75 years of age in the four trials comparing non-vitamin K oral anticoagulants to vitamin K antagonist for stroke prevention in atrial fibrillation. HR, hazard ratio; NOAC, non-vitamin K oral anticoagulants; RR, relative risk; SSE, stroke or systemic embolism; VKA, vitamin K antagonist. Definition of major bleeding according to study criteria as well as dosing regimens is given for all studies in Table 1

The RE-LY trial compared the direct thrombin-inhibitor dabigatran to warfarin. In the overall trial, dabigatran at 2*150 mg/day reduced the rate of stroke or systemic embolism by 34% with a similar rate of major bleeds; dabigatran at 2*110 mg/day showed a similar rate of stroke or systemic embolism but reduced the rate of major bleeding by 20% compared with VKA [15]. As there was no clinical dose reduction criteria and both doses had been randomised to VKA in a 1:1:1 fashion, age did not influence dabigatran dosing. While dabigatran 2*150 mg/day reduced stroke or systemic embolism in the elderly by 33%, major bleeding was increased by 18%. While the bleeding increase did not reach statistical significance, the European Medicines Agency restrained the approval to patients < 80 years of age. On dabigatran 2*110 mg/day, the reduction of stroke and systemic embolism by 12% was not significant, but the risk of bleeding was similar to VKA (Figs. 1 and 2 top). Overall, in patients ≥ 75 years of age, the combined risk of stroke or systemic embolism and major bleeding was similar between VKA and both dosages of dabigatran [20]. While there was a clinical net benefit for both doses of dabigatran in the overall RE-LY trial, this conclusion cannot be extrapolated to patients ≥ 75 years of age. For these patients, the increase in major bleedings counteracts the benefit of lower embolic rates.

### Rivaroxaban in ROCKET-AF

The ROCKET-AF trial compared the factor Xa-inhibitor rivaroxaban to warfarin. In the overall trial, rivaroxaban was similarly effective for prevention of stroke or systemic embolism with a similar rate of major bleeds [16]. In ROCKET-AF 44% of patients were ≥ 75 years of age [21]. As the dose of rivaroxaban was only reduced in patients with impaired renal function, age did not directly influence rivaroxaban dosing. In the elderly, rivaroxaban was at least as effective preventing stroke or systemic embolism and similarly safe regarding major bleeding (Figs. 1 and 2 middle). In line with the overall ROCKET-AF trial, there was no clinical net benefit for patients ≥ 75 years of age for rivaroxaban over VKA.

### Apixaban in ARISTOTLE

The ARISTOTLE trial compared the factor Xa-inhibitor apixaban to warfarin. In the overall trial, apixaban compared with VKA reduced the rate of stroke or systemic embolism by 21% and the rate of major bleeding by 31% [17]. In ARISTOTLE 31% of patients were ≥ 75 years of age [22]. Age directly influenced apixaban dosing as age ≥ 80 years was one of three dose reduction criteria, of which two had to be present to reduce the daily dose by 50%. While only 5% of the total trial population fulfilled at least two dose reduction criteria, 95% of those patients were ≥ 75 years of age. In the elderly, apixaban reduced stroke or systemic embolism by 29% and major bleeding by 36% (Figs. 1 and 2 middle). Similar to the overall ARISTOTLE trial, the clinical net benefit for patients ≥ 75 years of age was in favour of apixaban by reducing both embolic as well as major bleeding events.

### Edoxaban in ENGAGE-AF

The ENGAGE-AF trial compared the factor Xa-inhibitor edoxaban to warfarin. This article will only focus on the higher tested dose of edoxaban (full dose of 60 mg with clinical dose reduction criteria to 30 mg), because another lower dosing regimen of 30/15 mg edoxaban had been tested in another 7034 patients in an original 1:1:1 randomisation, but was not approved for stroke prevention in AF. In the overall trial, edoxaban was equally effective in preventing stroke or systemic embolism (non-significant reduction by 13%) and reduced major bleeding by 20% compared with VKA [14]. In ENGAGE-AF 40% of patients were ≥ 75 years of age [23]. Age did not directly influence edoxaban dosing. While 25% of the total trial population fulfilled dose reduction criteria, 65% of those patients were ≥ 75 years of age. In the elderly, edoxaban was at least as effective preventing stroke or systemic embolism and reduced major bleeding by 17% (Figs. 1 and 2 bottom). Similar to the overall ENGAGE-AF trial, the clinical net benefit for patients ≥ 75 years of age was in favour of edoxaban driven primarily by reducing major bleeding.

In summary, it seems reasonable to use NOACs for elderly patients with AF instead of VKA. Similar to the general AF populations investigated in the trials, all four NOACs are at least as effective as VKA. There appears to be some additional benefit for apixaban and the higher dose of dabigatran regarding stroke prevention. However, on the bleeding side, the higher dose of dabigatran is not recommended in patients ≥ 80 years of age by several authorities. Overall, apixaban and edoxaban had a lower risk of major bleeding in elderly patients as in the general study populations.

When comparing NOACs in general to VKA, the major advantage of NOACs is the 52% risk reduction for intracranial haemorrhage, and the major downside is the 25% increase in gastrointestinal bleedings. However, in all four NOAC approval studies, there was only an excess of 160 gastrointestinal bleedings for 221 intracranial haemorrhages prevented on NOAC versus VKA [24]. Nevertheless, both bleeding sites need to be addressed in the elderly population.

## Gastrointestinal Bleedings on NOACs in the Elderly

**Fig. 3:**
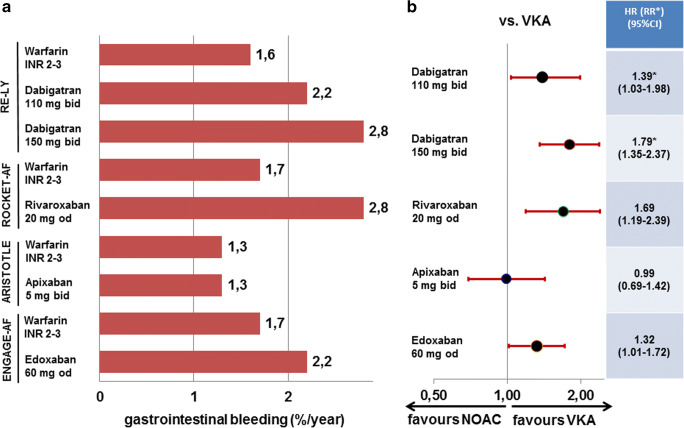
Event rates (**a**) and hazard ratios (or relative risk for dabigatran) (**b**) for gastrointestinal bleeding in patients ≥ 75 years of age in the four trials comparing non-vitamin K oral anticoagulants to vitamin K antagonist for stroke prevention in atrial fibrillation. HR, hazard ratio; NOAC, non-vitamin K oral anticoagulants; RR, relative risk; VKA, vitamin K antagonist. Dosing regimens are given for all studies in Table 1

In patients ≥ 75 years of age, gastrointestinal bleeding rates are increased on some NOACs compared with VKA (dabigatran 110 mg + 38%, dabigatran 150 mg + 75%, rivaroxaban + 69%, edoxaban + 29%) [20, 21, 23]. The analysis of patients aged ≥ 75 years on apixaban does not state gastrointestinal bleeding rates [22], but the submission document to German health authorities shows an equal risk of major gastrointestinal bleedings for patients ≥ 75 years of age for apixaban compared with VKA in the approval study [25](Fig. 3). However, it is difficult to attribute the risk solely to the specific NOAC in the trial. The risk for gastrointestinal bleeding may also depend on co-administration of platelet inhibitors (increase of bleeding) and proton-pump inhibitors (decrease of bleeding). In RE-LY, 40% of all patients received concomitant acetylsalicylic acid, and 18% received proton-pump inhibitors or a histamine 2 receptor antagonist [20]. In ROCKET-AF, acetylsalicylic acid was used at randomisation by 57% of patients and 14% received proton-pump inhibitors. In ARISTOTLE, similarly 18% received gastric antacid drugs, but acetylsalicylic acid was only used in about 20% of patients throughout the trial [26]. In ENGAGE-AF, the ratio of patients receiving acetylsalicylic acid at randomisation was similar in patients aged ≥ 75 years with 29% as in the overall trial population; dedicated information on proton-pump inhibitors has not been mentioned in the publications [23]. Thereby, the lower rate of acetylsalicylic acid users in ARISTOTLE might have positively influenced the lower gastrointestinal bleeding signal. Nevertheless, the observation of lower gastrointestinal bleedings on apixaban compared with other NOACs is reported from several US and German post market healthcare databases [27, 28]. Of note, in a randomised placebo-controlled trial for low-dose anticoagulation in cardiovascular disease routine use of proton-pump inhibitors in patients receiving low-dose anticoagulation and/or aspirin, pantoprazole significantly reduced bleeding of gastroduodenal lesions [29].

In summary, it might be feasible to combine oral anticoagulation in particular in elderly patients with routine PPI treatment to prevent gastrointestinal bleeding and to refrain from concomitant antiplatelet therapy whenever possible.

## Intracranial Bleedings on NOACs in the Elderly

**Fig. 4:**
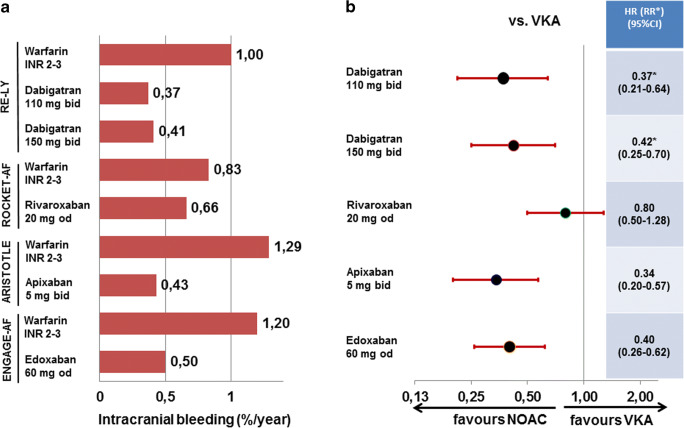
Event rates (**a**) and hazard ratios (or relative risk for dabigatran) (**b**) for intracranial haemorrhage in patients ≥ 75 years of age in the four trials comparing non-vitamin K oral anticoagulants to vitamin K antagonist for stroke prevention in atrial fibrillation. HR, hazard ratio; NOAC, non-vitamin K oral anticoagulants; RR, relative risk; VKA, vitamin K antagonist. Dosing regimens are given for all studies in Table 1

**Fig. 5:**
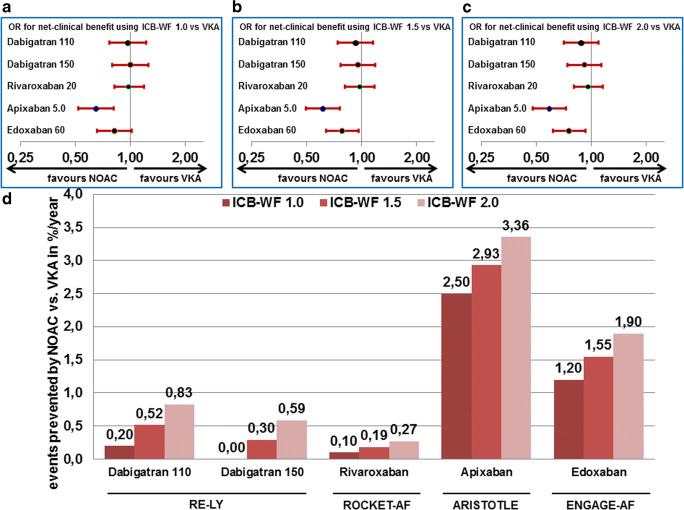
Net clinical benefit by NOACs compared with VKA in patients ≥ 75 years of age in the four NOAC approval trials for stroke prevention in atrial fibrillation based on the event rates for stroke or systemic embolism, major bleeding, and intracranial bleeding (ICB) expressed as odds ratios (OR) calculated based on a weighting factor for ICB, the ICB-WF, set at 1.0 for ICB having similar fatality than other events (**a**), at 1.5 for ICB having 50% higher fatality than other events (**b**), and at 2.0 for ICB having double the fatality than other events (**c**) or as number of events prevented/100 patient years of treatment; calculation of the net clinical benefit has been modified according to [18] as explained in the main text. NOAC, non-vitamin K oral anticoagulants; VKA, vitamin K antagonist

When assessing intracranial haemorrhages in patients ≥ 75 years of age, the rates with all NOACs are lower than in their respective matched VKA groups, but the effect appears to be less striking in the ROCKET-AF trial using rivaroxaban (Fig. 4). Previously, in a net clinical benefit analysis from the ATRIA trial [18], a higher factor was attributed to the risk of intracranial haemorrhage due to its higher fatality compared with other major bleedings and stroke or systemic embolism. In their publication, the authors defined the core net clinical benefit of anticoagulation therapy in AF as the annualised rate of thromboembolic events prevented minus the annualised rate of major bleedings induced, whereby intracranial haemorrhages are multiplied by a weighting factor. The weighting factor reflects the relative impact, in terms of death and disability, of an intracranial haemorrhage while receiving anticoagulation versus experiencing an ischaemic stroke while not receiving anticoagulation. In our analysis, we used the same principle to compare the different NOACs to the former standard of VKA. In their initial analysis, the authors used a weighting factor of 1.5 reflecting outcomes in the ATRIA study cohort [18]. When applying such an analysis for NOACs vs. VKA, one detects the highest and significant benefit in elderly patients compared with VKA on apixaban followed by edoxaban, whereas in elderly patients, rivaroxaban or either dose of dabigatran only provided a slight numerical benefit (Fig. 5). Including specifically intracranial bleeding in balancing risk and benefit in elderly anticoagulated AF patients, the overall impression favouring apixaban and to some extent edoxaban is not changed but indeed strengthened.

## NOAC Dose Reduction in the Elderly

For dabigatran, age had not been a dose reduction criterion in RE-LY [15]. Based on the higher bleeding rates, dabigatran 150 mg is not recommended for patients ≥ 80 years of age in Europe, and a lower dose should be considered for patients 75–79 years of age. In the USA, no age restriction is recommended.

For rivaroxaban, age had not been a dose reduction criterion in ROCKET-AF [16]. Dose reductions are not recommended for any age and are driven by renal function, which is more often impaired in elderly.

**Fig. 6:**
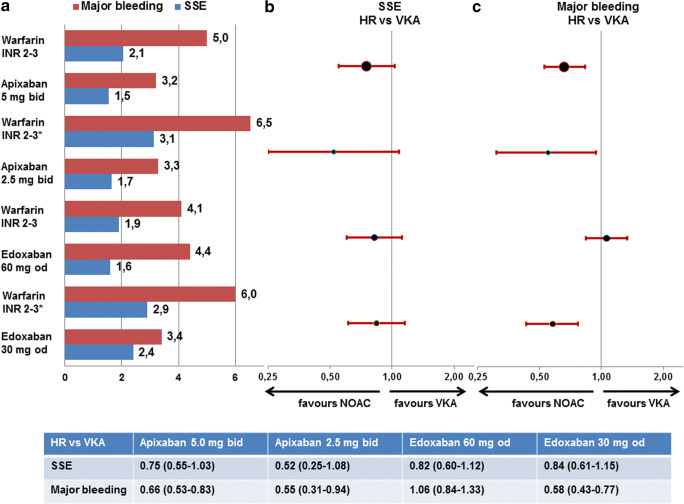
Event rates (**a**) and hazard ratios (**b**, **c**) for stroke or systemic embolism (**a**, **b**) and major bleedings (**b**, **c**) in patients ≥ 75 years of age in ARISTOTLE and ENGAGE-AF depending on the presence or absence of study-specific dose reduction criteria. HR, hazard ratio; NOAC, non-vitamin K oral anticoagulants; SSE, stroke or systemic embolism; VKA, vitamin K antagonist. Definition of major bleeding according to study criteria as well as dosing regimens is given for all studies in the main text. * indicates a group of patients qualifying for the study-specific dose reduction criteria who were treated with warfarin

For apixaban, age ≥ 80 years is one of the factors for dose reductions to 2*2.5 mg/day as recommended from ARISTOTLE [17] if two or more of them are met. Patients on VKA who meet these criteria have higher embolic and bleeding rates than non-affected VKA patients. Patients ≥ 75 years not meeting these criteria have a clinical net benefit compared with VKA similar to younger patients if they are treated with the full 2*5 mg/day dose of apixaban. Patients ≥ 75 years fulfilling the reduction criteria have a clinical net benefit compared with VKA if they are treated with the reduced 2*2.5 mg/day dose of apixaban (Fig. 6 top). Regarding the very elderly patients ≥ 80 years of age, there was a 30% clinical net benefit in favour of apixaban compared with VKA based on lower stroke or systemic embolism (1.5%/year vs. 1.9%/year) and major bleedings (3.6%/year vs. 5.4%/year) [22].

For edoxaban, age per se had not been a dose reduction criterion in ENGAGE-AF [14]. Dose reductions to 1*30 mg/day are recommended in clinical practice based on non-age-specific criteria. Patients on VKA who met the study criteria for dose reduction had higher embolic and bleeding rates than patients on VKA without these criteria. Patients ≥ 75 years receiving full dose (1*60 mg/day) edoxaban and not meeting these criteria had a similar clinical net benefit compared with patients with VKA as younger patients. Patients ≥ 75 years who fulfilled the reduction criteria and were treated with the reduced 1*30 mg/day dose of edoxaban had a clinical net benefit compared with VKA (Fig. 6 bottom). Regarding the very elderly patients ≥ 80 years of age, there was a 22% clinical net benefit in favour of edoxaban compared with VKA based on both lower embolic and major bleeding events [23].

In patients with dose reduction criteria, both apixaban and edoxaban demonstrate clinical net benefit. Again, the need for dose reduction does not change the overall interpretation of NOAC data in elderly patients but rather seems to have contributed to the positive profile of the two agents demonstrating best clinical net benefit.

In general, elderly patients with AF are not threatened or endangered by treatment with NOACs. First, there was no NOAC trial with lower efficacy than VKA; in contrast some strategies were even more effective. Second, in none of the trials did a NOAC cause more intracranial bleeds than VKA. Third, regarding prevention of extracranial major bleedings, there seems to be a difference between NOACs, but selection of the two strategies with clinically driven dose reduction criteria using apixaban and edoxaban. This is clearly visible once the overall clinical net benefit is assessed, irrespective of adding incremental weighting for intracranial bleeds. To prevent the potential downside of gastrointestinal bleedings, concomitant antiplatelet treatment should be avoided and proton-pump inhibitors considered in elderly anticoagulated patients.

## NOACs in Dual Antithrombotic Therapy Following Coronary Interventions in the Elderly

When a patient with AF requires PCI with consecutive DAPT, combining ASA + OAC (previously restricted to VKA) + a P2Y_12_ inhibitor (in combinations predominantly clopidogrel) can increase the risk of bleeding by three- to fourfold compared with OAC alone [30]. Therefore, there had been a quest for alternatives in antithrombotic combination therapies with less bleeding risk. Therefore, two strategies were pursued more recently: first, choosing a NOAC instead of VKA might reduce the bleeding risk already as all four NOAC strategies tested in and approved for stroke prevention in AF use an antithrombotic intensity that is comparable to sub-therapeutic dosing of low-molecular weight heparins or VKA, and, second, dropping one out of three antithrombotic drugs early after PCI might further reduce bleeding risk, if it is safe regarding prevention of stent thrombosis. VKA and all four NOACs have now been investigated in differing trials combining anticoagulants with antiplatelet treatment aiming for mostly early dropping of acetylsalicylic acid, but some have also applied lower doses of anticoagulants than actually tested and approved for stroke prevention in atrial fibrillation. The only trial randomising for acetylsalicylic acid did so on average ~ 7 days after PCI. All trials included much less patients than the approval trials for stroke prevention, so their primary endpoint was not efficacy in terms of stroke (and other ischaemic events) prevention, but rather the reduction of major or clinically relevant non-major bleedings. Thereby, dropping mostly acetylsalicylic acid in the NOAC dual antithrombotic treatment arm will automatically lead to a lower primary bleeding endpoint, but this self-fulfilling prophecy does not mean that patients are equally protected from embolic events. Therefore, we focus first on whether there was a signal on non-equal anti-ischaemic protection compared with conventional triple therapy and second whatever data has been reported from elderly patients, which in some studies was ≥ 75 years and in others ≥ 80 years of age. The different trials and definition of the primary endpoint are displayed in Table 2.

**Table 2:** Characteristics of trials combining anticoagulants and platelet inhibitors after percutaneous coronary interventions

	WOEST [31]	PIONEER-AF [32]	RE-DUAL PCI [33]	AUGUSTUS [34]	ENTRUST-AF [35]
Triple antithrombotic treatment	VKA INR 2–3 + clopidogrel + acetylsalicylic acid	VKA INR 2–3 + P2Y_12_ inhibitor + acetylsalicylic acid	VKA INR 2–3 + P2Y_12_ inhibitor + acetylsalicylic acid	VKA INR 2–3 + P2Y_12_ inhibitor	VKA INR 2–3 +P2Y_12_ inhibitor +acetylsalicylic acid
Dual antithrombotic treatment	VKA INR 2–3 + clopidogrel	Rivaroxaban 15 mg + P2Y_12_ inhibitor	Dabigatran 150 mg + P2Y_12_ inhibitor	Apixaban 5 mg + P2Y12 inhibitor	Edoxaban 60 mg + P2Y_12_ inhibitor
Additional arm	–	Rivaroxaban 2*2.5 mg + acetylsalicylic acid + P2Y_12_ inhibitor	Dabigatran 110 mg* + P2Y_12_ inhibitor	Second randomisation ± acetylsalicylic acid	–
Patients (*n*)	563	2124	2725	4614	1506
Mean age (years)	70.3	70	69/72*	71	70
≥ 75 years (≥ 80 years^#^)	394 (70%)	717 (34%)	450 (17%)^#^	689 (14%)^#^	506 (34%)
Definition of primary bleeding endpoint	**↓**	**↓**	**↓**	**↓**	**↓**
	*TIMI major and non-major* [37]*:* • Any intracranial bleeding • Spontaneous gross haematuria or hematemesis (> 120 ml), even if the haemoglobin or haematocrit drop was less than 3 g/dL or less than 10% • Unobserved loss ≥ 4 g/dl in haemoglobin or ≥ 12% in haematocrit		*ISTH major and clinically-relevant non-major* [38, 39]*:* • Fatal bleeding • Symptomatic bleeding in a critical area or organ such as intracranial, intraspinal, intraocular, retroperitoneal, intra-articular or pericardial, or intramuscular with compartment syndrome • Bleeding causing a fall in haemoglobin level ≥ 2 g/dL or leading to transfusion ≥ 2 units of whole blood or red cells • Any sign or symptom of haemorrhage (e.g. more bleeding than would be expected for a clinical circumstance, including bleeding found by imaging alone) that does not fit the criteria above but does meet at least one of the following criteria: - Requiring medical intervention by a healthcare professional - Leading to hospitalisation or increased level of care - Prompting a face to face (i.e. not just a telephone or electronic communication) evaluation		

Strategies, definition of bleeding events, and proportion of patients with age ≥ 75 years in the five trials comparing oral anticoagulants in dual and triple antithrombotic regimens following percutaneous coronary intervention and/or acute coronary syndromes in patients with indication for anticoagulation. VKA, vitamin K antagonist; *in RE-DUAL patients ≥ 80 years outside the USA (≥ 70 years in Japan) were only randomised to either VKA or dabigatran 110 mg; ^#^PIONEER-AF and RE-DUAL PCI trials only report data for patients ≥ 80 years

The WOEST trial investigated the combination of a vitamin K antagonist with clopidogrel following PCI in anticoagulated patients (69% anticoagulated because of AF). In patients ≥ 75 years of age, the rate of clinically significant bleedings was similarly reduced as in the overall cohort (p-interaction 0.9157). In the overall cohort, major adverse cardiovascular events were even reduced on OAC + clopidogrel compared with conventional triple therapy; however, a dedicated analysis regarding elderly patients is not feasible based on the rather low overall study size [31].

The PIONEER-AF trial investigated the combination of the factor Xa-inhibitor rivaroxaban (in a lower than approved dose of 1*15 mg/day during combination antithrombotic treatment or 1*10 mg/day in case of impaired renal function) with mostly clopidogrel following PCI in AF patients. In patients ≥ 75 years of age, the rate of clinically significant bleedings was reduced by 35%; however, major adverse cardiovascular events increased by 69%. A dedicated analysis regarding major bleedings in patients ≥ 75 years is not yet published; however, their reduction in the overall cohort was 36%. The major concern regarding comorbid patients in PIONEER-AF is that patients with prior stroke or TIA had not been included in the trial and a lower dose of rivaroxaban than recommended and approved for stroke prevention in AF had been used. The increase in ischaemic events was particularly observed in the elderly [32].

The RE DUAL-PCI trial investigated the combination of the direct thrombin inhibitor dabigatran (2*110 mg/day for all patients ≥ 80 years of age outside the USA (≥ 70 for Japan)). In patients ≥ 80 years of age, the rate of clinically significant bleedings was reduced by 29%, but the rate of major adverse cardiovascular and revascularisation events increased by 18%. As the trials are underpowered for ischaemic event rates, one has to be careful interpreting non-significant increases in potential harm. A dedicated analysis regarding major bleedings in patients ≥ 80 years is not yet published. The major concern regarding comorbid patients in RE DUAL-PCI is that only the lower of the approved doses was used as per approval outside the USA, which was associated with an increased rate for stroke, myocardial infarction, and stent thrombosis in the overall cohort [33].

The AUGUSTUS trial investigated the combination of the factor Xa-inhibitor apixaban (in the approved dose of 2*5 mg/day or 2*2.5 mg/day in case of dose reduction criteria as in ARISTOTLE [17]) with mostly clopidogrel following PCI and/or an acute coronary syndrome in AF. In patients ≥ 80 years of age, the rate of clinically relevant bleeding was 15% lower on apixaban compared with VKA and 83% higher on acetylsalicylic acid compared with placebo. The rate for all-cause mortality and ischaemic events was 16% lower on apixaban compared with VKA and 24% lower on acetylsalicylic acid compared with placebo; however, in the overall trial, this was attributable to the VKA cohort only. Event rates and statistical comparisons for patients ≥ 80 years of age have not yet been published [34].

The ENTRUST-AF PCI trial investigated the combination of the factor Xa-inhibitor edoxaban (in the approved dose of 1*60 mg/day or 1*30 mg/day in case of dose reduction criteria as in ENGAGE [14]) with mostly clopidogrel following PCI in AF. In patients ≥ 75 years of age, the rate of clinically relevant bleeding was 17% lower on edoxaban compared with VKA, other event rates and statistical comparisons for patients ≥ 75 years of age have not yet been published [35].

In summary, it seems reasonable for elderly patients with AF, who require PCI, to administer acetylsalicylic acid peri-procedurally followed by OAC plus clopidogrel. OAC can principally mean NOAC, but limitations apply to the currently published studies with dabigatran (more ischaemic events in lower dose, only lower dose for elderly patients) and rivaroxaban (excluding patients with higher stroke risk, dose reduction without proven clinical efficacy). Data for apixaban and edoxaban do suggest that full-dose NOAC instead of VKA is safe and efficient; however, final conclusions about the duration of acetylsalicylic acid co-medication for the elderly cannot be given at the moment similar to the unresolved status of that matter in the overall population. Current ESC recommendations emphasise that if NOACs are used, they should be given without empiric dose reduction [36]. The advantages of all combinations of NOAC and clopidogrel are significantly lower bleeding rates compared with conventional triple therapy using VKA. Another important contribution to prophylaxis of bleeding especially for elderly patients is to avoid permanent combinations of platelet inhibitors with (N)OAC for coronary artery disease and AF following the usual duration of DAPT or additive P2Y_12_ inhibition [1, 36].

## Conclusion

A frequent challenge for therapeutic strategies in elderly patients is their under-representation in clinical trials; thus, trial results cannot be universally extrapolated to patients ≥ 75 years of age. In general, elderly patients with AF profit from OAC compared with acetylsalicylic acid. Due to the increasing bleeding risk, acetylsalicylic acid monotherapy is no alternative for stroke prevention in AF in the elderly. OAC should be used based on the better efficacy at similar bleeding risk. In the four approval trials for NOACs in stroke prevention in AF, the percentage of patients ≥ 75 years of age ranged from 30 to 44%, which allows to assess this subgroup regarding their embolic and bleeding risk. Analysing the elderly subgroups of the NOAC trials demonstrates that these patients are actually not harmed by NOACs, but that there are two tested strategies which even show an additional clinical net benefit for apixaban or edoxaban. If elderly patients do meet the predefined dose reduction criteria for apixaban or edoxaban, they have lower embolic and bleeding risks compared with VKA. If they do not meet the predefined dose reduction criteria for apixaban or edoxaban, they still have a clinical net benefit compared with VKA on the higher dose of apixaban. If combined antithrombotic therapy is required after PCI, acetylsalicylic acid should only be given peri-procedurally, and until further studies become available, the NOAC dose approved for stroke prevention in AF should be used without empiric dose reduction in most patients; however, individual decisions have to be made in the very old. At the moment, the full effective doses of apixaban and edoxaban have shown at least a similar safety profile regarding bleeding compared with VKA.
